# The effectiveness of the laid-back position on lactation-related nipple problems and comfort: a meta-analysis

**DOI:** 10.1186/s12884-021-03714-8

**Published:** 2021-03-24

**Authors:** Zhi Wang, Qiuyue Liu, Lihua Min, Xiaorong Mao

**Affiliations:** 1 School of Medicine, University of Electronic Science and Technology of China, Chengdu, Sichuan China; 2 School of Nursing, North Sichuan Medical College, Nanchong, Sichuan China; 3 Department of Nursing, Sichuan Provincial People’s Hospital, University of Electronic Science and Technology of China, Chengdu, Sichuan China

**Keywords:** Breastfeeding, Biological nurturing, Laid-back breastfeeding, Meta-analysis

## Abstract

**Background:**

The importance of breastfeeding for maternal and child health is agreed upon worldwide. However, lactation-related nipple problems are common and are important factors affecting breastfeeding. Multiple studies recommended laid-back breastfeeding, but they are of various levels of quality, and the results are inconclusive.

**Methods:**

We systematically searched the following twelve databases from inception to January 28,2020: Cochrane Library, EMBASE, Medline, Ovid, PubMed, Web of Science, CINAHL, Scopus, Chinese National Knowledge Infrastructure (CNKI), China Biology Medicine disc (CBM), WanFang, and VIP. All studies regarding laid-back breastfeeding or biological nurturing were considered, regardless of whether they were randomized controlled trials. Two trained investigators independently evaluated the quality of the selected articles and screened the data. All the data were analysed separately using Review Manager Version 5.3 and STATA/SE Version 15.1.

**Results:**

A total of 12 studies involving 1936 groups of postpartum women and their newborns were included. The results of the meta-analysis showed that nipple pain (RR = 0.24; 95% CI 0.14, 0.40; *p* < 0.00001), nipple trauma (RR = 0.47; 95% CI 0.29, 0.75; *p* = 0.002) and correct latching position (RR = 1.22; 95% CI 1.11, 1.33; *p* < 0.0001) in the experimental groups were all better than those of the control groups, and the differences were statistically significant (*p* < 0.05), which indicates that the laid-back position has a positive effect on maternal breastfeeding. However, the results of position comfort showed that there was no statistical significance between the two groups (ES = 0.09; 95% CI -0.63, 0.81; *p* = 0.798).

**Conclusion:**

Compared with traditional breastfeeding positions, the laid-back position has been proven to be related to a decreased incidence of nipple pain and nipple trauma and is seemingly conducive to the use of the correct latching position. It is suggested that the laid-back position is helpful in solving lactation-related nipple problems and can be recommended as a position for breastfeeding. However, no significant difference in position comfort was found between the two groups based on the current evidence, and further studies are still needed to validate these results due to the limitations of the included studies.

**Supplementary Information:**

The online version contains supplementary material available at 10.1186/s12884-021-03714-8.

## Background

Breastmilk provides all the energy and nutrients that the infant needs for the first months of life, and it continues to provide up to half or more of a child’s nutritional needs during the second half of the first year and up to one third during the second year of life [1]. Two studies published in the journal *Lancet* [2, 3] showed that the lives of over 820,000 of children younger than 5 years of age could be saved every year if all children aged 0–23 months were optimally breastfed. In 2012, the World Health Assembly (WHA) Resolution 65.6 endorsed a comprehensive implementation plan on maternal, infant and young child nutrition, which specified that by 2025, the percentage of exclusive breastfeeding in the first 6 months should be increased to a target of up to at least 50% [4]. According to data from the World Health Organization (WHO), only approximately 44% of infants aged 0–6 months worldwide were exclusively breastfed over the period of 2015–2020 [1].

There are many factors that affect breastfeeding, among which the most important is the breastfeeding position. Inappropriate breastfeeding positions may have a negative effect on the mother’s wellbeing and exacerbate related diseases through a negative impact on the infant’s positioning and latching and the duration of breastfeeding events [5]. According to a study [6], approximately 70.3% of mothers suffered from breastfeeding difficulties, including cracked nipples, the perception of an insufficient amount of milk, pain, and fatigue. It is estimated that 80–90% of mothers experience nipple pain [7], and 58% of them experience nipple damage [8]. Nipple pain has been described by the mothers as sore nipples during and after breastfeeding [9], and it is the second most common reason for early weaning [10]. Nipple trauma is a macroscopic traceable cutaneous lesion in the area of the nipple and areola that may occur in the form of fissures, eroded skin and ulcerations, or clinical evidence of erythema, oedema, blisters, white, yellow, or dark stains, and ecchymosis [11]. The major causes of nipple pain and trauma are inappropriate breastfeeding techniques and improper infant positioning [10]. Nipple pain caused by an incorrect latching position is a common problem among breastfeeding mothers, which can lead to nipple trauma and pose an important obstacle to successful breastfeeding [12–14]. Poor latching is associated with pain when breastfeeding. In contrast, correct positioning and latching—in which the infant’s gum line is placed well over the mother’s lactiferous sinuses, the tongue is positioned under the areola, and both lips are flanged outward—are essential for increasing milk supply and intake [15–17]. Education regarding correct latching and infant positioning can lead to a decrease in nipple pain and an increased duration of breastfeeding [18].

There are various breastfeeding positions adopted by mothers. Traditional breastfeeding positions include the cradle, cross-cradle, side-lying and football positions, which are mostly dominated by the mother, ignoring the baby’s instincts and needs. Breastfeeding initiation is associated with the release of inborn baby reflexes and instinctive mothering behaviours [19]. Biological Nurturing (BN), which was developed by Colson in the early 1990s [20] and is also known as laid-back breastfeeding (LBBF), refers to the placement of the mothers in a comfortable, semi-reclined positions where every part of the body is supported, especially the shoulders, neck and arms, while the baby lies prone or on the stomach and their bodies not flat but tilted up in the process of breastfeeding [21]. BN is a breastfeeding concept that revolves around a return to biology and includes lactation concepts related to the environment, reflex, intervention, and neurodevelopment. It is defined as a neurobehavioural approach to the initiation of breastfeeding to reduce latching problems and the accidental early cessation of breastfeeding [19]. Biological nurturing can be used throughout the breastfeeding period (from the time of birth to the end of breastfeeding). It is a collective term for mother-baby positions and states that interrelate and interact to release primitive neonatal reflexes and spontaneous maternal breastfeeding behaviours [21].

However, at present, the varied quality of associated studies on biological nurturing has led to controversial results. The laid-back position has not been popularized in breastfeeding health education around the world, and few high-quality studies have been performed to serve as a backbone for this approach with regard to the effects of breastfeeding. The goal of this paper was to evaluate the effect of the laid-back position (biological nurturing) on breastfeeding through evidence-based methods to provide references for the formulation and specification of breastfeeding position(s).

## Methods

The study was prepared according to the Preferred Reporting Items for Systematic Review and Meta-Analysis (PRISMA) guidelines [22] (see Additional file 1). All articles were imported into to a citation manager (EndNote X9), and duplicates were removed. Two trained investigators (the first and second authors of this paper) searched the databases and screened the titles and abstracts independently.

### Inclusion and exclusion criteria

The criteria for studies to be included in this review were as follows: (1) participant groups included an experimental group, in which the mothers adopted a laid-back breastfeeding (biological nurturing) position, and a control group, in which any traditional breastfeeding position, including the cradle, cross-cradle, side-lying and football positions, were used; (2) the effects of the intervention were assessed as the incidence of nipple pain, nipple trauma, correct latching position and position comfort; and (3) a clinical study design, including randomized controlled trials (RCTs) and quasi-randomized controlled trials (Q-RCTs), was used.

﻿ Studies were excluded if they (1) were not focused on the effectiveness of the laid-back position on lactation-related nipple problems and comfort; (2) had no full text available; (3) reported unextractable or unrelated raw data and the authors could not be contacted; (4) were published other than in English or Chinese; and (5) were reviews, editorials, books, theses, news, etc.

### Search strategy

We systematically searched the following twelve databases from inception to January 28,2020: Cochrane Library, EMBASE, Medline, Ovid, PubMed, Web of Science, CINAHL, Scopus, Chinese National Knowledge Infrastructure (CNKI), China Biology Medicine disc (CBM), WanFang and VIP. The search was carried out using the following keywords or medical subject headings: [“breast-feed” OR “Feeding, Breast” OR “Breastfeeding” OR “Breast Feeding, Exclusive” OR “Exclusive Breast Feeding” OR “Breastfeeding, Exclusive” OR “Exclusive Breastfeeding” OR “Breast-feeding”] AND [“Laid-back” OR “Half lay” OR “Semi recumbent position” OR “Semi-reclining position” OR “semirecumbent” OR “Half lying type” OR “semi supine position” OR “semiprone position”] OR [“Biological Nurturing” OR “recommending biological breeding” OR “Laid-back Breastfeeding” OR “Laid-back breast feeding” OR “Half lay breast-feeding”]. To obtain a full understanding of this topic, we also manually tracked the references in the included articles and contacted investigators in the field to locate unpublished studies, but none were available. The search strategies are listed in Additional file 2.

### Data extraction and synthesis

Two reviewers independently assessed the studies for eligibility and extracted the data using a standardized data extraction form, which was then checked by the third reviewer. Disagreements were resolved via discussion with the third author. Studies selected for inclusion were transferred to a Microsoft Excel spreadsheet for extraction of data items of: basic information from the included literature (first author, year of publication, study design), baseline characteristics (sample size, inclusion and exclusion criteria, delivery mode, maternal category, gestational weeks, age mothers) and analysis indexes (interventions, intervention time, outcomes). When information regarding the study methods and results was unclear, we contacted the authors for further details. Disagreements were resolved by discussion with all members of the research team until a consensus was reached.

### Quality assessment

The risk of bias (RoB) of each RCT was evaluated independently by two investigators using the RoB 2.0 tool obtained from the Cochrane Handbook for Systematic Reviews of Interventions (Version 62,019) [23], which included the following domains: bias arising from the randomization process, bias due to deviations from intended interventions, bias due to missing outcome data, bias in measurement of the outcome, and bias in selection of the reported result. For each domain, the tool comprises a series of “signalling questions”, and once they were answered, the next step was to reach a risk-of-bias judgement and assign one of three levels to each domain [23, 24]: “low risk of bias”, “some concerns”, or “high risk of bias”. Finally, risk-of-bias judgements within domains were mapped to an overall judgement for the outcome. The risk of bias of each quasi-randomized controlled trial was evaluated independently by two investigators using the JBI Critical Appraisal Checklist for Quasi-Experimental Studies (JBI, 2016) [25] by assigning “Yes”, “No”, “Unclear” or “Not applicable” for each of the 9 items. Any disagreements regarding the inclusion of studies were resolved through discussion; if a consensus could not be reached, a senior reviewer served as the final arbiter.

### Statistical analysis

Statistical analyses were performed with Review Manager Version 5.3 and STATA/SE Version 15.1 (StataCorp, College Station, TX, USA). To eliminate man-made errors and combine the functions of the different software programs, all the data were analysed separately by two investigators using different programs (ZW: STATA/SE and QL: RevMan). Dichotomous outcome data were pooled using the risk ratio (RR) and presented as the 95% confidence interval (CI), and continuous outcome data are presented as the mean ± standard deviation (SD) and were pooled using the mean difference (MD) and 95% CI. For data for the same outcome presented in some studies as dichotomous data and in other studies as continuous data (for example, position comfort), we re-expressed the odds ratios as standard mean differences (SMDs) according to the simple formula SMD = logor×(√3/π = 0.5513), computing them (or the log odds ratios) and their standard errors for all studies in the meta-analysis and allowing dichotomous and continuous data to be combined by using the generic inverse-variance method [26, 27].

Heterogeneity was assessed statistically by using the Chi^2^ (χ^2^, or chi-squared) test and the I^2^ statistic. When *p* > 0.10 or I^2^ ≤ 50%, the results of the associated studies were said to be homogenous or to have acceptable heterogeneity, and a fixed-effects model was utilized. When *p* ≤ 0.10 or I^2^ > 50%, it was considered that there was heterogeneity in the results of the multiple included studies, and subgroup analysis or sensitivity analysis was performed to identify the sources of heterogeneity. Then, the selected studies were removed one by one, and the overall correlation results and I^2^ were recalculated. A random-effects model was selected if the heterogeneity could not be eliminated. To assess the effects of covariates on the pooled estimates, subgroup analysis and meta-regression analysis were conducted [28, 29]. If there was considerable variation in the results that could not be removed, the meta-analysis was abandoned, and the evidence was presented in a narrative form only.

Publication bias was detected using Egger’s linear regression test [30] since no more than 10 original articles were enrolled in any analysis. *p*-values (two–tailed) < 0.05 were considered statistically significant. For studies with publication bias, we conducted sensitivity analyses (trim and fill method) to explore the publication bias and the robustness of the meta-analysis conclusions to different assumptions about the causes of the funnel plot asymmetry [31–35].

## Results

### Search results

**Fig. 1:**
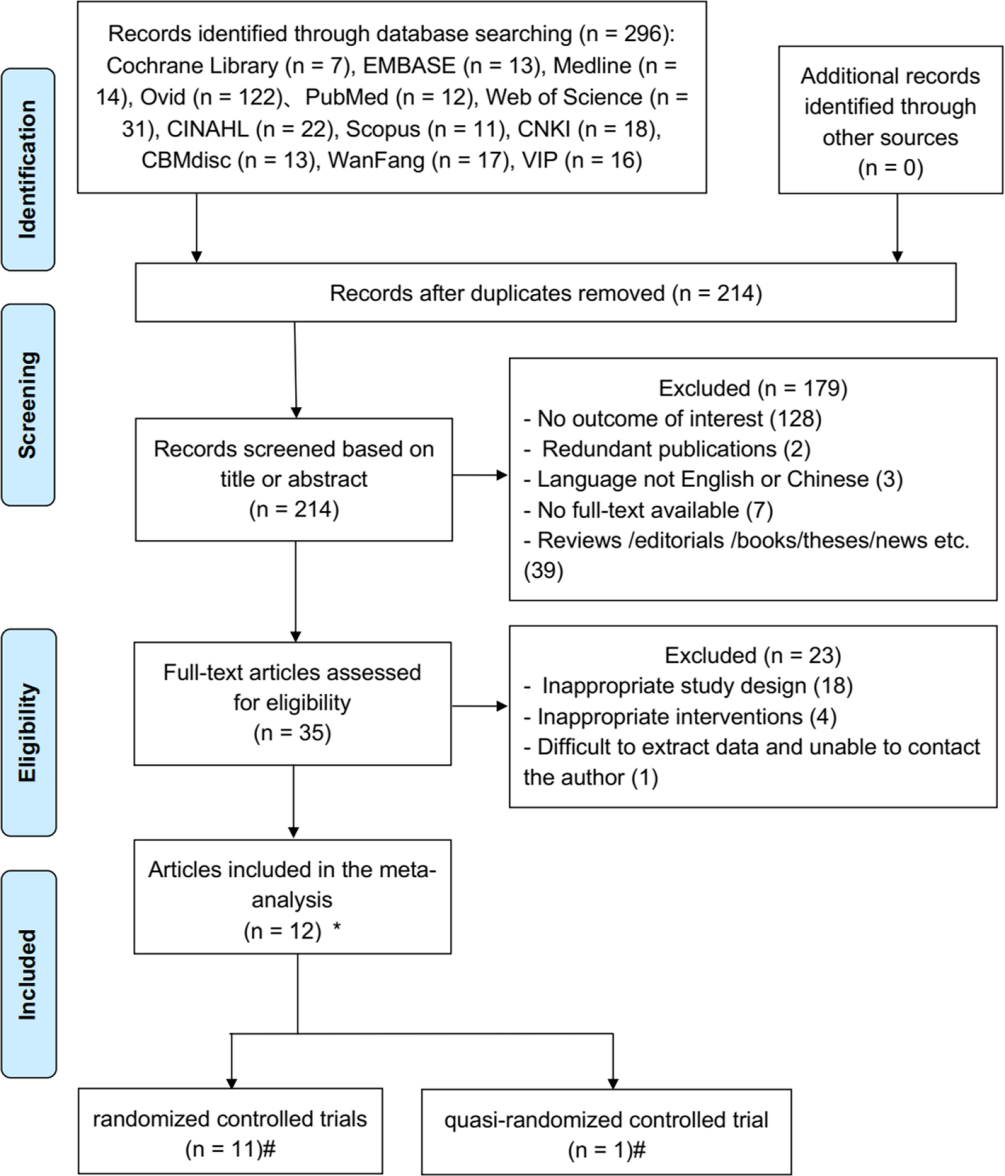
PRISMA flow diagram of the literature search and study selection. *Twelve papers were included in meta-analysis. #Eleven studies were included in RCTs, and one study was a Q-RCT

The literature selection process is shown in the PRISMA flow diagram [22] (Fig. 1), including the reasons for exclusion. Initially, a total of 296 publications were retrieved from the following 12 electronic databases: Cochrane Library (*n* = 7), EMBASE (*n* = 13), Medline (*n* = 14), Ovid (*n* = 122), PubMed (*n* = 12), Web of Science (*n* = 31), CINAHL (*n* = 22), Scopus (*n* = 11), CNKI (*n* = 18), CBM (*n* = 13), WanFang (*n* = 17) and VIP (*n* = 16). A search of the reference lists of the included studies yielded no additional studies. After eliminating duplicates, 214 references were included. Then, the remaining 214 studies were screened through their titles and abstracts, excluding an additional 179 articles. The remaining 35 articles were screened through their full texts, of which 22 were excluded because they were not appropriate study designs (*n* = 18) or interventions (*n* = 4). Moreover, one article was excluded because the data were difficult to extract, and we were unable to contact the author. Finally, 12 articles fulfilled the eligibility criteria and were included in the meta-analysis.

### Characteristics of the included studies

Twelve studies met the inclusion criteria, including eleven RCTs and one quasi-randomized controlled trial and consisting of 1936 participants (970 in the experimental groups and 966 in the control groups). The included studies were reported in English (one trial) and Chinese (eleven trials). No significant difference was observed between the two groups in terms of the studied variables. All the included studies were published from 2017 to 2019. The intervention period of the included studies lasted from 3 days to 8 weeks. The age of the infants was estimated to be 0–3 days according to the starting time of the intervention. The outcomes included nipple trauma (*n* = 7), nipple pain (*n* = 8), correct latching position (*n* = 3) and position comfort (*n* = 4). We established three subgroups based on intervention, delivery mode and maternal category. Studies were divided into two groups according to whether they implemented skin-to-skin care (SSC) [36]; in other words, whether the mother and baby had direct skin contact. In the subgrouping based on intervention, the studies were divided into the “LBBF” group and the “LBBF+SSC” group according to whether the skin-to-skin care was performed on the basis of laid-back breastfeeding. In the subgrouping based on maternal delivery mode, the studies were divided into the “vaginal delivery” group and the “vaginal delivery & caesarean” group. In the subgrouping based on maternal category, the studies were divided into the “primipara” group and the “primipara & multipara” group according to whether the delivery times were restricted. Further details about the included studies are shown in Table 1.

**Table 1:** Characteristics of the studies included in the meta-analysis

Author (year)	Sample size(*n*) (E/C)	Study design	Vaginal delivery/Caesarean (*n*)		Primipara/Multipara (*n*)		Gestational weeks (Mean ± SD) (E/C)	Age of mother (years) (Mean ± SD) (E/C)	Intervention (E/C)	Age of infant	Intervention period	Outcomes
			E	C	E	C						
Zhuang (2019) [37]	75/75	RCT	38/37	35/40	–	–	–	26.2 ± 4.7/ 25.3 ± 4.5	Laid-back breastfeeding (BN) /Traditional breastfeeding position	0 day	1-8w	Nipple pain
Shi et al. (2017) [38]	84/84	RCT	50/34	52/32	56/28	55/29	36–39/ 36–40	27.98 ± 4.25/ 28.51 ± 4.69	Laid-back breastfeeding (BN) /Traditional breastfeeding position	–	1-3d	Nipple pain, Nipple trauma
Li et al. (2017) [39]	100/100	RCT	64/36	68/32	60/40	64/36	38–40/ 37–40	27.86 ± 4.25/ 27.43 ± 4.17	Laid-back breastfeeding (BN) /Traditional breastfeeding position	<3d	1-3d	Nipple pain, Nipple trauma, Position Comfort, Correct latching position
Yu et al. (2019) [40]	100/100	RCT	0/100	0/100	100/0	100/0	39.06 ± 1.08/ 39.33 ± 1.06	27.43 ± 4.14/ 27.40 ± 3.81	Laid-back breastfeeding (BN) /Cradle breastfeeding (Traditonal position)	0 day	1-3d	Nipple trauma, Position Comfort
Zeng et al. (2019) [41]	60/60	RCT	42/18	44/16	–	–	39.35 ± 1.23/39.42 ± 1.13	26.75 ± 4.23/ 27.28 ± 4.42	Laid-back breastfeeding (BN) /Cradle breastfeeding (Traditonal position)	0 day	1d-4w	Nipple pain
Puapornpong et al. (2017) [42]	76/76	RCT	0/76	0/76	–	–	38.5 ± 0.9/38.6 ± 1.0	27.5 ± 5.9/ 27.1 ± 6.1	Laid-back breastfeeding (BN) /Side-Lying Breastfeeding (Traditonal position)	0 day	1d-6w	Position Comfort
Liu et al. (2019) [43]	49/49	RCT	–	–	–	–	–	–	Laid-back breastfeeding (BN) /Traditional breastfeeding position	–	1-4w	Nipple pain, Nipple trauma
Zhang (2019) [44]	74/70	RCT	58/16	56/14	74/0	70/0	38.89 ± 0.96/ 39.09 ± 0.97	25.82 ± 3.43/ 26.16 ± 3.62	Laid-back breastfeeding (BN + SSC) /Cradle breastfeeding (Traditonal position)	0 day	1d-6w	Nipple pain, Correct latching position
Zhao (2019) [45]	48/48	RCT	48/0	48/0	–	–	39.57 ± 1.06/ 39.40 ± 1.11	26.31 ± 2.82/ 27.27 ± 3.83	Laid-back breastfeeding (BN + SSC) /Traditional breastfeeding position	0 day	1-3d	Nipple pain
Liang et al. (2017) [46]	200/200	RCT	200/0	200/0	–	–	–	–	Laid-back breastfeeding (BN + SSC) /Traditional breastfeeding position	0 day	1-3d	Nipple trauma
Wang (2019) [47]	50/50	RCT	27/23	30/20	34/16	32/18	–	27.9 ± 2.3/ 27.7 ± 2.1	Laid-back breastfeeding (BN + SSC) /Standard care (Traditonal position)	0 day	0-1 m	Nipple pain, Nipple trauma
Lu et al. (2019) [48]	54/54	Q-RCT	–	–	26/28	25/29	38.95 ± 0.45/ 39.50 ± 0.50	26.88 ± 3.32/ 26.76 ± 3.25	Laid-back Breastfeeding (BN + SSC) /Standard care (Traditonal position)	0 day	0-1w	Nipple trauma, Position Comfort, Correct latching position

*E* experimental group, *C* control group, *h* hour, *d* day, *w* week, *m* month, *SD* standard deviation

### Risk of bias assessment

**Fig. 2:**
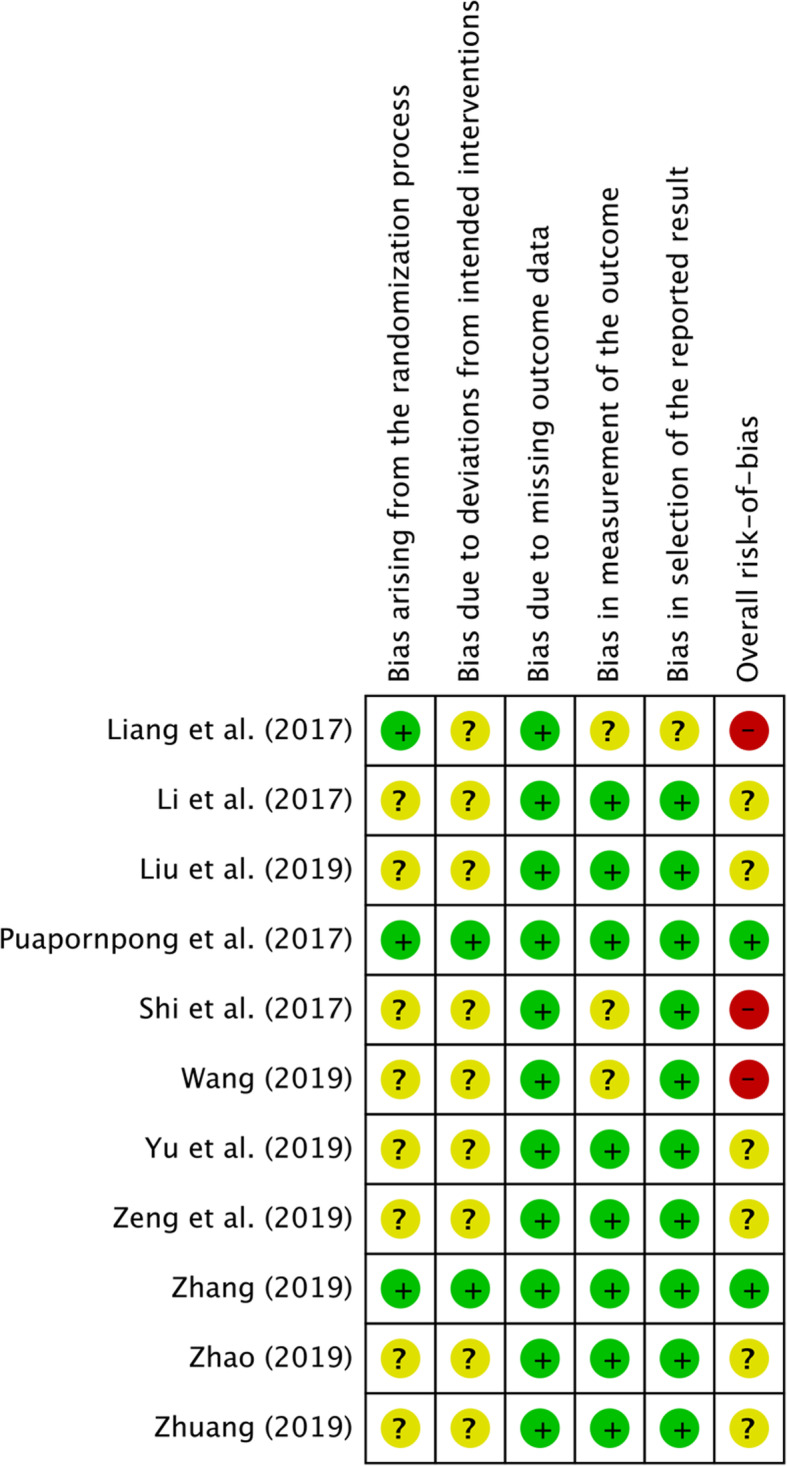
Risk of bias summary graph (RCT)

The quality of all included RCT studies is shown in Table 2 and Fig. 2. For the judgement of “bias arising from the randomization process”, five [37–41] of the 11 included RCTs did not show detailed information on the random components in the sequence generation process, eight studies [37–44] were judged to raise some concerns, and three studies [45–47] were judged to have a low risk of bias. They concealed the allocation sequence with opaque envelopes that were sequentially numbered and sealed with a tamper-proof seal. With regard to the judgement of “bias due to deviations from intended interventions”, only two studies [46, 47] reported compliance with the intervention, and they were judged to have a low risk of bias. All included studies were judged to be at low risk of bias in the judgement of “bias due to missing outcome data”. For the judgement of “bias in measurement of the outcome”, three studies [39, 41, 45] were judged to raise some concerns, and the others [37, 38, 40, 42–44, 46, 47] were judged to have a low risk of bias. For the judgement of “bias in selection of the reported result”, one study [45] was judged to have raised some concerns because the trial did not perform the analysis in accordance with a pre-specified plan, and ten studies [37–44, 46, 47] were judged to have a low risk of bias. Overall, three RCTs (27%) had a high RoB, six (55%) showed some concerns, and two (18%) had a low RoB for their outcomes. The quality of the one quasi-randomized controlled trial included is presented in Table 3.

**Table 2:** Results of critical appraisal for Randomized Controlled Trials (Cochrane, 2019)

Study (year)	Bias arising from the randomization process	Bias due to deviations from intended interventions	Bias due to missing outcome data	Bias in measurement of the outcome	Bias in selection of the reported result	Overall risk-of-bias
Zhuang (2019) [37]	Some concerns	Some concerns	Low risk	Low risk	Low risk	Some concerns
Shi et al. (2017) [38]	Some concerns	Some concerns	Low risk	Some concerns	Low risk	High risk
Li et al. (2017) [39]	Some concerns	Some concerns	Low risk	Low risk	Low risk	Some concerns
Yu et al. (2019) [40]	Some concerns	Some concerns	Low risk	Low risk	Low risk	Some concerns
Zeng et al. (2019) [41]	Some concerns	Some concerns	Low risk	Low risk	Low risk	Some concerns
Puapornpong et al. (2017) [42]	Low risk	Low risk	Low risk	Low risk	Low risk	Low risk
Liu et al. (2019) [43]	Some concerns	Some concerns	Low risk	Low risk	Low risk	Some concerns
Zhang (2019) [44]	Low risk	Low risk	Low risk	Low risk	Low risk	Low risk
Zhao (2019) [45]	Some concerns	Some concerns	Low risk	Low risk	Low risk	Some concerns
Liang et al. (2017) [46]	Low risk	Some concerns	Low risk	Some concerns	Some concerns	High risk
Wang (2019) [47]	Some concerns	Some concerns	Low risk	Some concerns	Low risk	High risk

**Table 3:** Results of critical appraisal for the quasi-experimental studies (JBI, 2016)

Questions (potential bias)	Lu et al. (2019) [48]
1. Is it clear in the study what is the ‘cause’ and what is the ‘effect’? (causation/reverse causation)	Yes
2. Were the participants included in any comparisons similar? (selection bias)	Yes
3. Were the participants included in any comparisons receiving similar treatment/care, other than the intervention of interest? (history threat/systematic difference/contamination bias)	Yes
4. Was there a control group? (measurement bias)	Yes
5. Were there multiple measurements of the outcome both before and after the intervention? (maturation threat, regression to the mean)	Yes
6. Was follow-up complete, and if not, was follow-up adequately reported and strategies to deal with loss to follow-up employed? (attrition bias)	Yes
7. Were the outcomes of participants included in any comparisons measured in the same way? (instrumentation/testing effects threats)	Yes
8. Were outcomes measured in a reliable way? (detection/instrument/measurement bias)	Yes
9. Was appropriate statistical analysis used? (performance / detection bias)	Yes

### Synthesis of results

#### Nipple pain

**Fig. 3:**
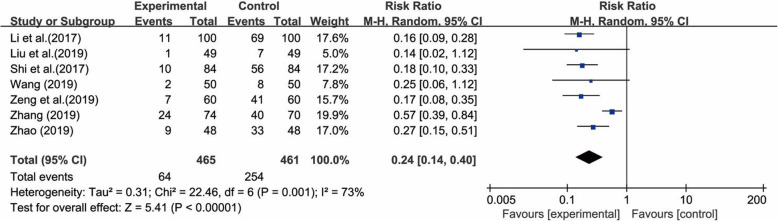
Forest plot for the incidence of nipple pain between the laid-back position and the traditional position in breastfeeding

Eight studies [37–39, 41–44, 46], with a total of 1076 pairs of postpartum women and their newborns, compared traditional breastfeeding positions vs. laid-back breastfeeding in terms of nipple pain. There was substantial heterogeneity among these studies (χ^2^ = 125.27, *p* < 0.00001, I^2^ = 94%) according to the heterogeneity test. It seems that one outlier [43] had a substantial influence on the results of the overall meta-analysis according to sensitivity analyses and was thus a major source of the heterogeneity. After removing it, the result of the heterogeneity test with the remaining 7 studies decreased I^2^ to 73% (χ^2^ = 22.46, *p* = 0.001, I^2^ = 73%). Therefore, a random-effects model was utilized for the meta-analysis, and the results showed that the experimental group had a lower incidence of nipple pain than the control group (RR = 0.24; 95% CI 0.14, 0.40; *p* < 0.00001) (Fig. 3). It is worth mentioning that the result of the heterogeneity test decreased I^2^ to 0% (χ^2^ = 2.02, *p* = 0.85, I^2^ = 0%) after removing the two studies [43, 46], probably because of the different ﻿measurement tools used in the included studies.

#### Nipple trauma

**Fig. 4:**
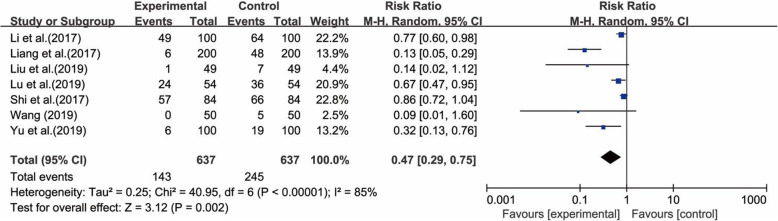
Forest plot for the incidence of nipple trauma between the laid-back position and the traditional position in breastfeeding

Seven studies [37–41, 45, 48], which included 1274 pairs of postpartum women and their newborns, reported nipple trauma. The results of the heterogeneity test for these studies showed that there was substantial heterogeneity (χ^2^ = 40.95, *p* < 0.00001; I^2^ = 85%), probably because of the different interventions used. However, the sensitivity analyses did not show any study to substantially influence the heterogeneity. Thus, a random-effects model was utilized for the meta-analysis, and the results showed that the experimental group had a lower incidence of nipple trauma than the control group (RR = 0.47; 95% CI 0.29, 0.75; *p* = 0.002) (Fig. 4).

#### Correct latching position

**Fig. 5:**
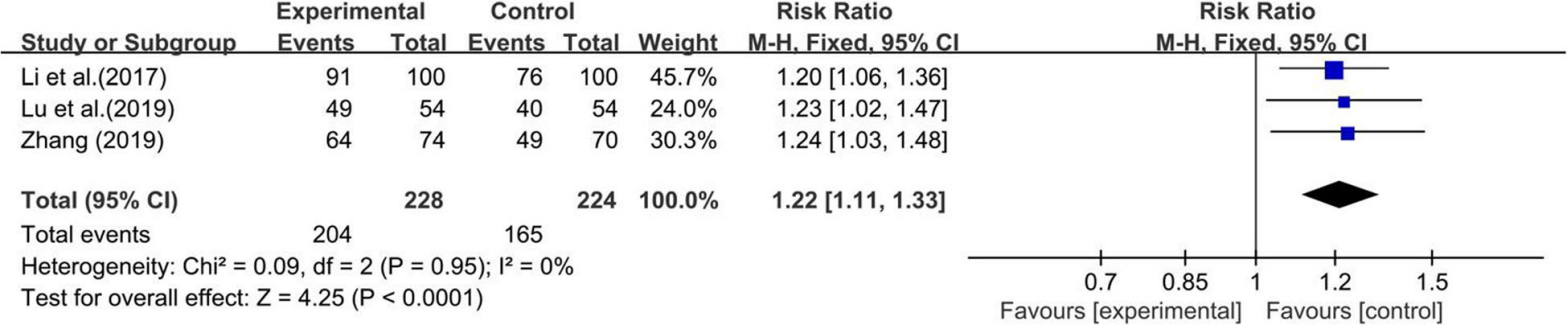
Forest plot for the correct latching position between the laid-back position and the traditional position in breastfeeding

Three studies [38, 46, 48], which included 452 pairs of postpartum women and their newborns, reported on the correct latching position. The incidence of using the correct position for latching did not significantly differ between the two groups (χ^2^ = 0.09, *p* = 0.95, I^2^ = 0%), so we used a fixed-effect model to pool the summary outcome. The results showed that the experimental group had a higher incidence of using the correct position for latching than the control group (RR = 1.22; 95% CI 1.11, 1.33; *p* < 0.0001) (Fig. 5).

#### Position comfort

**Fig. 6:**
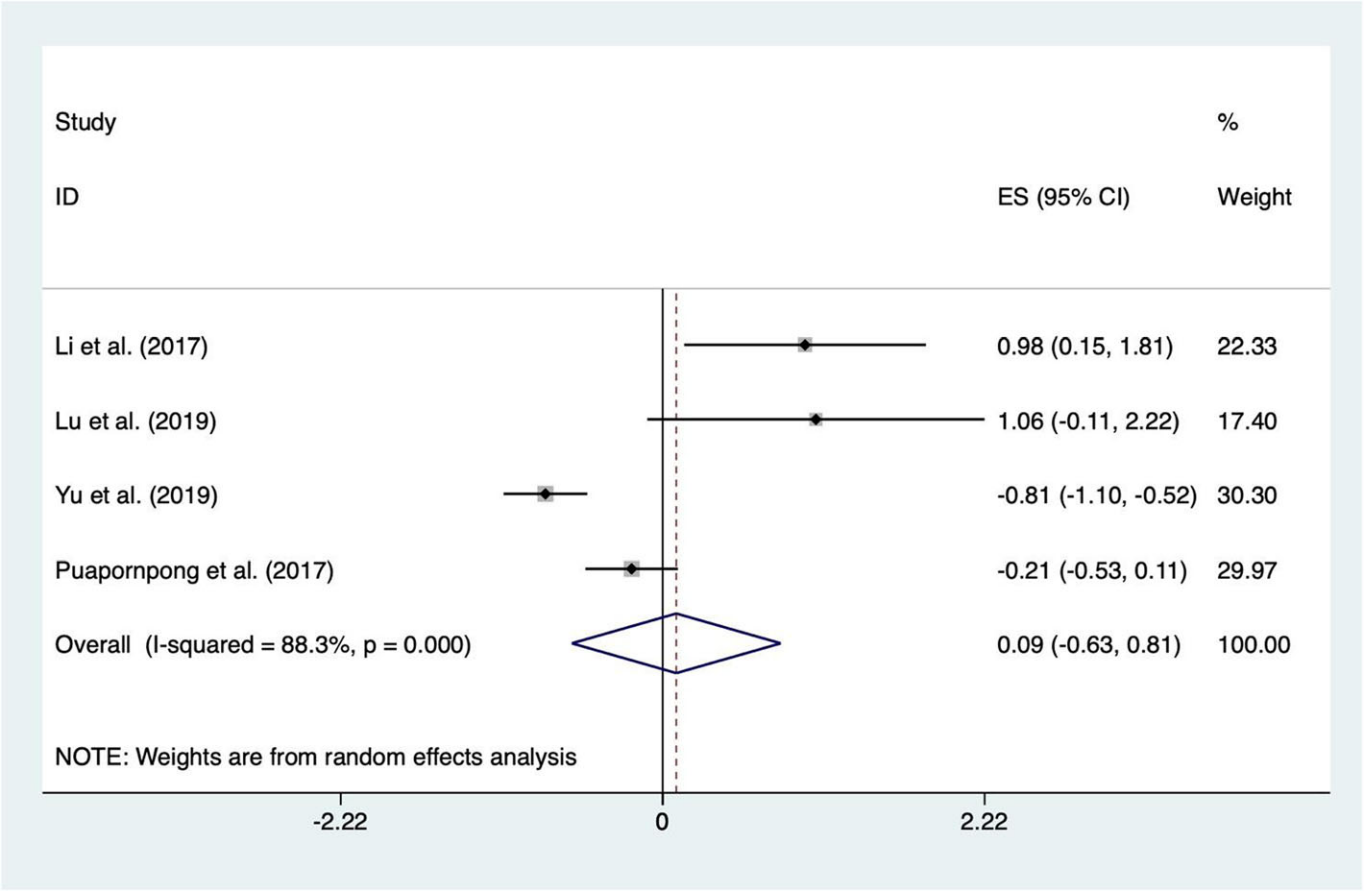
Forest plot for the position comfort between the laid-back position and the traditional position in breastfeeding

Four studies [38, 40, 47, 48] reported data on position comfort, and these studies included 660 pairs of postpartum women and their newborns. There were two dichotomous data [38, 48] and two continuous data [40, 47] among them. The results of the heterogeneity test showed that there was substantial heterogeneity among these studies (χ^2^ = 25.58, *p* < 0.0001, I^2^ = 88%) using the heterogeneity test, probably because of the different delivery modes included in the study. Nevertheless, the sensitivity analyses did not show any study to be substantially influence the heterogeneity. Therefore, a random-effects model was utilized for the meta-analysis, and the results showed that there was no statistical significance between the two groups (ES = 0.09; 95% CI -0.63, 0.81; *p* = 0.798) (Fig. 6).

#### Subgroup analysis

Three subgroup analyses were undertaken based on the intervention, delivery mode and maternal category. We evaluated the reliability of the outcomes, and the results are presented in Tables 4 and 5. For the outcome of nipple trauma, the results of the subgroup analysis were, on the whole, the same as the overall results, and the direction did not change. There was no evidence of a different effect related to the intervention (*p* for interaction = 0.24), delivery mode (*p* for interaction = 0.37), or maternal category (*p* for interaction = 0.37). The subgroup analysis for the different interventions showed that the experimental group had a lower incidence (RR = 0.68; 95% CI 0.47, 0.99) of nipple trauma than the control group among subjects who performed LBBF, but the difference was not significant (RR = 0.24; 95% CI 0.04, 1.31) for the group that performed both skin-to-skin care and LBBF+SSC. The subgroup analysis for the different delivery modes and maternal categories showed that the vaginal delivery & caesarean (RR = 0.50, 95% CI 0.30, 0.82) and primipara & multipara (RR = 0.50, 95% CI 0.30, 0.82) groups both had a slightly higher incidence of nipple trauma than the caesarean (RR 0.32; 95% CI 0.13, 0.76) and primipara (RR = 0.32; 95% CI 0.13, 0.76) groups, respectively. Similarly, the results of the subgroup analyses for nipple pain indicate that there was no evidence of a different effect related to the intervention (*p* for interaction = 0.51), delivery mode (*p* for interaction = 0.97), and maternal category (*p* for interaction = 0.14). Overall, the results of the subgroup analysis showed that all the experimental groups had a lower incidence of nipple trauma than the control groups, indicating no change in the results of the study.

**Table 4:** Subgroup Analysis of the Effect of Intervention Elements on Nipple Trauma

Subgroups	Number of studies	﻿Participants (n)	Heterogeneity test outcomes		Effects model	Meta-analysis	﻿Interaction *p*-value
		﻿(Experimental/Control)	*p*-value	I^2^ (%)		RR (95% CI)	
Intervention							0.24
LBBF	4 [38–40, 43]	333/333	0.023	68.5%	Random	0.68 (0.47, 0.99)	
LBBF +SSC	3 [46–48]	304/304	0.000	90.2%	Random	0.24 (0.04, 1.31)	
Deliver mode							0.37
Caesarean	1 [40]	100/100	–	–	Random	0.32 (0.13, 0.76)	
Vaginal delivery & caesarean	6 [38, 39, 43, 46–48]	537/537	0.000	85.8%	Random	0.50 (0.30, 0.82)	
Maternal category							0.37
Primipara	1 [40]	100/100	–	–	Random	0.32 (0.13, 0.76)	
Primipara & multipara	6 [38, 39, 43, 46–48]	537/537	0.000	85.8%	Random	0.50 (0.30, 0.82)

**Table 5:** Subgroup Analysis of the Effect of Intervention Elements on Nipple Pain

Subgroups	Number of studies	Participants (n)	Heterogeneity test outcomes		Effects model	Meta-analysis	Interaction *p*-value
		(Experimental/Control)	*p*-value	I^2^ (%)		RR (95% CI)	
Intervention							0.51
LBBF	5 [37–39, 41, 43]	368/368	0.000	96.9%	Random	0.23 (0.06, 0.94)	
LBBF +SSC	3 [44, 45, 47]	172/168	0.096	57.3%	Random	0.39 (0.21, 0.71)	
Deliver mode							0.97
Vaginal delivery	1 [45]	48/48	–	–	Random	0.27 (0.15, 0.51)	
Vaginal delivery & caesarean	7 [37–39, 41, 43, 44, 47]	492/488	0.000	94.9%	Random	0.28 (0.12, 0.66)	
Maternal category							0.14
Primipara	1 [44]	74/70	–	–	Random	0.57 (0.39, 0.84)	
Primipara & multipara	7 [37–39, 41, 43, 45, 47]	466/466	0.000	95.6%	Random	0.24 (0.08, 0.70)	

#### Publication bias

We used Egger’s linear regression test to detect the publication bias arising from various influencing factors quantitatively because concern remains that visual interpretation of the funnel plots is inherently subjective. The *p*-values for the correct latching position (*p* = 0.152) and position comfort (*p* = 0.138) were greater than 0.05, which indicates that there is no significant publication bias. Although the *p*-values for nipple pain (*p* = 0.008) and nipple trauma (*p* = 0.013) were less than 0.05, there were no missing ‘counterparts’ to fill after the data were analysed by the trim and fill method. This indicates that the results of the two outcomes are stable and that the effect of publication biases is negligible. In summary, the results showed no significant risk of publication bias among the included studies.

## Discussion

This meta-analysis was conducted to estimate the effect of the laid-back position on lactation-related nipple problems. ﻿The results of this study showed that the experimental group had a lower incidence of nipple trauma (22.4% vs. 38.5%) and nipple pain (13.8% vs. 55.1%) than the control group. This suggests that the laid-back position has a positive effect on maternal breastfeeding with regard nipple pain, nipple trauma and the correct position of latching. Further study regarding position comfort remains to be conducted.

Nipple pain is reported as one of the main causes of abandoning breastfeeding prematurely [49, 50]. Most women experience some degree of pain during breastfeeding, ranging from mild to severe, which may be accompanied by nipple trauma. Our meta-analysis showed that the experimental group had a lower incidence of nipple pain than the control group (13.8% vs. 55.1%, RR = 0.24; 95% CI 0.14, 0.40; *p* < 0.00001). This result is similar to a study carried out in ﻿Italy [51], which reported that biological nurturing significantly reduced the risk of sore nipples from 46.9 to 27.8% (RR 0.59, 95% CI 0.40, 0.88). These results may be explained by a higher proportion of successful latching and self-attachment with the laid-back position [51]. Nipple pain was measured on a rating scale that was developed based on the characteristics of the general population. No unified and specialized comprehensive assessment scale for nipple pain has been formed.

Nipple trauma is the main cause of nipple pain, and it is a well-recognized risk factor for breastfeeding cessation [50]. Our results suggest that BN reduced the incidence of nipple trauma by 16.1% (RR = 0.47; 95% CI 0.29, 0.75; *p* = 0.002). Nipple trauma includes nipple redness and swelling, cracks, blisters, ulcers, keratinization and defects [46]. Nipple cracks were the most common type of nipple trauma in this study, and 4 studies [37, 40, 41, 45] showed that the laid-back position could help to reduce the incidence of nipple cracks compared with the traditional position (4.2% vs. 19.8%). ﻿Nipple trauma causes pain and discomfort, which render it difficult for the mother to continue breastfeeding [52]. Nipple pain and nipple trauma exert an influence on each other. An improper feeding position can interfere with the tissue repair process and can lead to further damage [12]. We should pay greater attention to the evaluation of these two aspects of breastfeeding.

Correction of positioning and latching is the most common experience-based recommendation for the treatment of nipple pain [53]. A qualitative analysis to identify breastfeeding barriers in the early postpartum period found that the most common barrier was the mother’s perception of an inadequate milk supply and difficulty with latching [54]. This study indicated that BN increased the success rate of “establishing the correct latching position” with an RR of 1.22 (95% CI 1.11, 1.33; *p* < 0.00001). The laid-back position is conducive to obtaining the correct position of latching (89.5% in the intervention group and 73.7% in the control group), which may contribute to successful breastfeeding. However, this conclusion should be treated with caution because only three trials [38, 46, 48] were included.

Position comfort in this study is regarded theoretically as a state of strengthening by having the needs of human experience met, which causes mothers to be happy with their health care in the process of breastfeeding [55]. It is unclear from this study whether the laid-back position is superior to the traditional position regarding comfort in the period of breastfeeding, because the available current evidence did not reveal a significant difference in position comfort between the two groups. This could be due to the small sample size of the included studies or the different types of data, which weakens the assessment of the results of the meta-analysis. Thus, additional research about the effect of the laid-back position on position comfort should be conducted in the future.

Breastfeeding is a biology-based nurturing method rooted in human instinct [21]. Laid-back breastfeeding can be adopted even if there is early separation after birth or the mother is suffering from problems with breastfeeding. The National Childbirth Trust (NCT) breastfeeding counsellor Ros Vinall [56] considers that biological nurturing or “laid-back breastfeeding” taps into the mothers’ and babies’ own instincts to successfully perform breastfeeding. She also highlighted that BN approach can remove breastfeeding from the medical model, with its need for instruction and prescriptive rules. Colson’s research emphasizes the biological underpinnings of breastfeeding, empowering parents to be active participants in feeding rather than merely relying on the instincts of the infant [21]. Laid-back breastfeeding is a revelation for human beings, as it accords with our humanist, non-interventionist and back-to-biological spirit.

The quality of the included studies was moderate, and the results should be interpreted with caution. Many of the original studies implemented a single-blind design. The nurses who followed up with the breastfeeding outcomes did not know the breastfeeding position groups, and none of the included studies reported whether the researchers who analysed data knew which was the experimental group. Few of the original studies reported compliance with the intervention. The reason might be that breastfeeding is a private activity, and researchers can only provide guidance, making it difficult to monitor the whole process. Thus, we should focus on this issue in future studies and take measures to ensure compliance, such as videotaping with informed consent.

All the data were analysed separately by two investigators using different software programs. The results show that this method can effectively avoid human errors such as data entry errors and improper operation, and it also combines the functions of the different programs. We found that, in practice, the I^2^ and Z values of the continuous variables obtained by the two software programs were slightly different, but this did not affect the outcomes, which is probably related to the algorithms used by the software; all other results from the programs were identical.

### Limitation

The present meta-analysis has some potential limitations: (1) We considered all RCTs and quasi-randomized controlled trials published in English and Chinese; studies published in other language were not included, leading to potential selection bias; (2) The heterogeneity for certain comparations was significant, which may have influenced the pooled results, despite our using a random-effects model; (3) Three subgroups analyses were performed according to intervention, delivery mode and maternal category, but other factors that could influence the outcomes might be present; and (4) In this study, only quantitative indicators were analysed, and the vast majority of the included studies were published in Chinese, because most of the studies obtained from the search were qualitative reports written in other countries.

## Conclusions

The results of this meta-analysis suggest that the laid-back position is helpful for solving lactation-related nipple problems and can be used as a recommended position for breastfeeding. Nurses and researchers may instruct mothers to assume the laid-back position to decrease the incidence of nipple pain and nipple trauma during breastfeeding. However, no significant difference in position comfort was found between the two groups based on the current evidence. Further high-quality and large-scale studies are needed to validate these results due to the limitations of the included studies.

## Supplementary Information

**Additional file 1.** PRISMA 2009 checklist.

**Additional file 2.** Search strategies.

## Data Availability

The data sets analyzed during the current study will be available upon reasonable request of the corresponding author.

## Abbreviations

CNKI: Chinese National Knowledge Infrastructure
WHA: World Health Assembly
WHO: World Health Organization
BN: Biological Nurturing
LBBF: Laid-back Breastfeeding
PRISMA: Preferred Reporting Items for Systematic Reviews and Meta-Analyses
RCT: Randomized Controlled Trial
Q-RCT: Quasi-randomized controlled trial
RoB: risk of bias
RR: Risk ratio
95% CI: 95% confidence interval
SD: Standard deviation
MD: Mean difference
SMDs: Standard mean differences
SSC: Skin-to-skin care
NCT: National Childbirth Trust
