# Supplementary material for: The effectiveness of the laid-back position on lactation-related nipple problems and comfort: a meta-analysis
Source: BMC Pregnancy Childbirth. 2021 Mar 24;21:248. doi: 10.1186/s12884-021-03714-8 (PMC7992330; doi:10.1186/s12884-021-03714-8)
Supplement: Supplementary file 2 — Additional file 2. Search strategies. [file 12884_2021_3714_MOESM2_ESM.docx]

Additional file 2: ﻿Search strategies.

1. Embase：

n=13

| Search | Query |
| --- | --- |
| #1 | ‘breast feed’:ti,ab,kw OR feeding,breast:ti,ab,kw OR breastfeeding:ti,ab,kw OR ‘breast feeding,exclusive’:ti,ab,kw OR ‘exclusive breast feeding’:ti,ab,kw OR breastfeeding,exclusive:ti,ab,kw OR ‘exclusive breastfeeding’:ti,ab,kw OR ‘breast feeding’:ti,ab,kw |
| #2 | ‘laid back’:ti,ab,kw OR ‘half lay’:ti,ab,kw OR ‘semi recumbent position’:ti,ab,kw OR ‘semi-reclining position’:ti,ab,kw OR semirecumbent:ti,ab,kw OR ‘half lying type’:ti,ab,kw OR ‘semi supine position’:ti,ab,kw OR ‘semiprone position’:ti,ab,kw |
| #3 | ‘biological nurturing’:ti,ab,kw OR ‘recommending biological breeding’:ti,ab,kw OR ‘laid-back breastfeeding’:ti,ab,kw OR ‘laid-back breast feeding’:ti,ab,kw |
| #4 | #1 AND #2 |
| #5 | #3 OR #4 |

2. Scopus：

n=11

[( ( TITLE-ABS-KEY ( "Biological Nurturing" )  OR  TITLE-ABS-KEY ( "recommending biological breeding" )  OR  TITLE-ABS-KEY ( "Laid-back Breastfeeding" )  OR  TITLE-ABS-KEY ( "Laid-back breast feeding" )  OR  TITLE-ABS-KEY ( "Half lay breast-feeding" ) ) )  OR  ( ( ( TITLE-ABS-KEY ( "breast-feed" )  OR  TITLE-ABS-KEY ( "Feeding,Breast" )  OR  TITLE-ABS-KEY ( "Breastfeeding" )  OR  TITLE-ABS-KEY ( "Breast Feeding,Exclusive" )  OR  TITLE-ABS-KEY ( "Exclusive Breast Feeding" )  OR  TITLE-ABS-KEY ( "Breastfeeding,Exclusive" )  OR  TITLE-ABS-KEY ( "Exclusive Breastfeeding" )  OR  TITLE-ABS-KEY ( "Breast-feeding" ) ) )  AND  ( ( TITLE-ABS-KEY ( "Laid-back" )  OR  TITLE-ABS-KEY ( "Half lay" )  OR  TITLE-ABS-KEY ( "Semi recumbent position" )  OR  TITLE-ABS-KEY ( "Semi-reclining position" )  OR  TITLE-ABS-KEY ( "recumbent" )  OR  TITLE-ABS-KEY ( "Half lying type" )  OR  TITLE-ABS-KEY ( "semi supine position" )  OR  TITLE-ABS-KEY ( "semiprone position" ) ) ) )](https://www.scopus.com/results/documentSpellSuggest.uri?sort=plf-f&src=s&mltEid=&mltAll=t&sid=f59c2cd64891e4af9cfe339afb257113&sot=comb&sdt=comb&sl=922&s=%28+%28+TITLE-ABS-KEY+%28+%22Biological+Nurturing%22+%29+OR+TITLE-ABS-KEY+%28+%22recommending+biological+breeding%22+%29+OR+TITLE-ABS-KEY+%28+%22Laid-back+Breastfeeding%22+%29+OR+TITLE-ABS-KEY+%28+%22Laid-back+breast+feeding%22+%29+OR+TITLE-ABS-KEY+%28+%22Half+lay+breast-feeding%22+%29+%29+%29+OR+%28+%28+%28+TITLE-ABS-KEY+%28+%22breast-feed%22+%29+OR+TITLE-ABS-KEY+%28+%22Feeding%2cBreast%22+%29+OR+TITLE-ABS-KEY+%28+%22Breastfeeding%22+%29+OR+TITLE-ABS-KEY+%28+%22Breast+Feeding%2cExclusive%22+%29+OR+TITLE-ABS-KEY+%28+%22Exclusive+Breast+Feeding%22+%29+OR+TITLE-ABS-KEY+%28+%22Breastfeeding%2cExclusive%22+%29+OR+TITLE-ABS-KEY+%28+%22Exclusive+Breastfeeding%22+%29+OR+TITLE-ABS-KEY+%28+%22Breast-feeding%22+%29+%29+%29+AND+%28+%28+TITLE-ABS-KEY+%28+%22Laid-back%22+%29+OR+TITLE-ABS-KEY+%28+%22Half+lay%22+%29+OR+TITLE-ABS-KEY+%28+%22Semi+recumbent+position%22+%29+OR+TITLE-ABS-KEY+%28+%22Semi-reclining+position%22+%29+OR+TITLE-ABS-KEY+%28+%22recumbent%22+%29+OR+TITLE-ABS-KEY+%28+%22Half+lying+type%22+%29+OR+TITLE-ABS-KEY+%28+%22semi+supine+position%22+%29+OR+TITLE-ABS-KEY+%28+%22semiprone+position%22+%29+%29+%29+%29&origin=resultslist)
